# 

**Published:** 1869-05

**Authors:** 


### CONTENTS:

### ORIGINAL COMMUNICATIONS.

ARTICLE I.—Review of Prof. F. G. Lemercier's Lecture on Clastic Anatomical Models. By Prof. Henry R. Noel, M.D. .......... 1

ARTICLE II.—Microscopy of the Dental Tissues. By S. P. Cutler, M.D., D.D.S. .......... 10

ARTICLE III.—Plaster as an Impression Material. By R. K. George, D.D.S. .......... 16

ARTICLE IV.—Notes from Dental Practice. CASE. I.—Treatment of Exposed Pulps .......... 22

CASE. II.—A Peculiar Form of Ulceration of the Gums 24

### CORRESPONDENCE.

ARTICLE V.—Letter from Dr. Thomas W. Evans of Paris .......... 25

### SELECTED ARTICLES.

ARTICLE VI.—On the Necessity of Artistic Knowledge and Critical Taste to Highest Success in the Dental Profession. By G. H. Perine, D.D.S. .......... 26
ARTICLE VII.—Good and Bad Teeth .......... 32
ARTICLE VIII.—Devitalizing the Dental Pulp, and Treatment Preparatory to Filling. By Dr. J. P. Wilson .......... 36
ARTICLE IX.—Dentine. By Prof. Henry S. Chase, M.D. .......... 38
ARTICLE X.—Some Unusual Phenomena attending Anæsthesia. By Frederick D. Lente, M.D. .......... 40

### MONTHLY SUMMARY.

Comminuted Fracture of Nasal Bones and Superior Maxilla; Sinking of Eyeball into Maxillary Sinus .......... 43
Dentistry in Japan .......... 44
Plaster Impressions .......... 44

### BIBLIOGRAPHICAL NOTICES.

Atlas to the Pathology of the Teeth .......... 45
The Life of the Trichina .......... 45

### EDITORIAL DEPARTMENT.

Salutatory .......... 46

Southern Dental Association .......... 47

Correction. Dr. Thos. W. Evans .......... 49

White's Dentifrices, &c .......... 50

Goodall's Elastic or Spring Plates .......... 50

